# Impacts of the active layer on runoff in an upland permafrost basin, northern Tibetan Plateau

**DOI:** 10.1371/journal.pone.0192591

**Published:** 2018-02-22

**Authors:** Tanguang Gao, Tingjun Zhang, Hong Guo, Yuantao Hu, Jianguo Shang, Yulan Zhang

**Affiliations:** 1 Key Laboratory of Western China’s Environmental Systems (Ministry of Education), College of Earth and Environmental Sciences, Lanzhou University, Lanzhou, China; 2 University of Arkansas, Fayetteville, Arkansas, United States of America; 3 State Key Laboratory of Cryospheric Science, Northwest Institute of Eco-Environment and Resources, Chinese Academy of Science, Lanzhou, China; Universidade de Vigo, SPAIN

## Abstract

The paucity of studies on permafrost runoff generation processes, especially in mountain permafrost, constrains the understanding of permafrost hydrology and prediction of hydrological responses to permafrost degradation. This study investigated runoff generation processes, in addition to the contribution of summer thaw depth, soil temperature, soil moisture, and precipitation to streamflow in a small upland permafrost basin in the northern Tibetan Plateau. Results indicated that the thawing period and the duration of the zero-curtain were longer in permafrost of the northern Tibetan Plateau than in the Arctic. Limited snowmelt delayed the initiation of surface runoff in the peat permafrost in the study area. The runoff displayed intermittent generation, with the duration of most runoff events lasting less than 24 h. Precipitation without runoff generation was generally correlated with lower soil moisture conditions. Combined analysis suggested runoff generation in this region was controlled by soil temperature, thaw depth, precipitation frequency and amount, and antecedent soil moisture. This study serves as an important baseline to evaluate future environmental changes on the Tibetan Plateau.

## Introduction

Understanding the interactions between changing hydrology and degrading permafrost is essential to reduce uncertainties in predicting the responses of water resources and aquatic ecosystems to climate change in high altitude/latitude regions [1–5]. Active layer hydrological processes and runoff generation in permafrost regions are well studied in the Arctic and sub-Arctic. For example, Carey and Woo [6] set up a two-layer flow system whereby most drainage occurred as quickflow in the porous organic layer, and as preferential flow in pipes, rills, and interconnected surface depressions in Wolf Creek, Yukon. In Scotty Creek of the Canadian sub-Arctic, permafrost thaw significantly influenced runoff by lowering the hydraulic gradient, thickening the active layer, and reducing the surface area of the drainage [7–8]. Johansson et al [9] studied the water balance in Western Greenland, and the interaction between the active layer, lake and talik. A 3-D modeling quantified the responses of different water flow and water storage components of terrestrial hydrology to shifts from the present climate landscape regime in Forsmark to a possible future Arctic periglacial landscape regime with or without permafrost [10].The lowering of the permafrost table and thinning seasonal freezing layer have resulted in creased soil storage capacity and allow the redistribution of later summer precipitation in Yenisei River [11].

Mountain permafrost areas, which constitute nearly two-thirds of the Tibetan Plateau, are the headwater source regions for several of Asia’s large rivers [12]. Active-layer thickness has increased, on average, by approximately 4.26 cm yr^-1^ along the Qinghai-Tibetan Railway from 2002 to 2012 [13]. Some studies have investigated the impact of permafrost thaw on the hydrology of the Tibetan Plateau. A negative water balance in the source region of the Yangtze, Yellow, and Lantsang Rivers in the northeast Tibetan Plateau was found to result from increased terrestrial evaporation and decreased river runoff [4, 14–15]. However, a number of studies have shown that streamflows in the Lhasa, Kunlun, and Heihe Rivers, located in the southern and northern Tibetan Plateau, have increased over the past decades [16–18].

Despite the importance of permafrost degradation on hydrology in the plateau, there is still a limited understanding of water flow and storage processes during permafrost thaw. These processes may influence runoff, and thus are needed to explain changing streamflow and capacity, and to predict flow variation in the future [19]. The previous studies attribute a deepening active layer and thawing permafrost as the cause of changes in hydrological regimes, but few have specifically examined the response of runoff processes and generation to active layer variation, particular in the particular research stations or small equipped watersheds.

**Fig 1:**
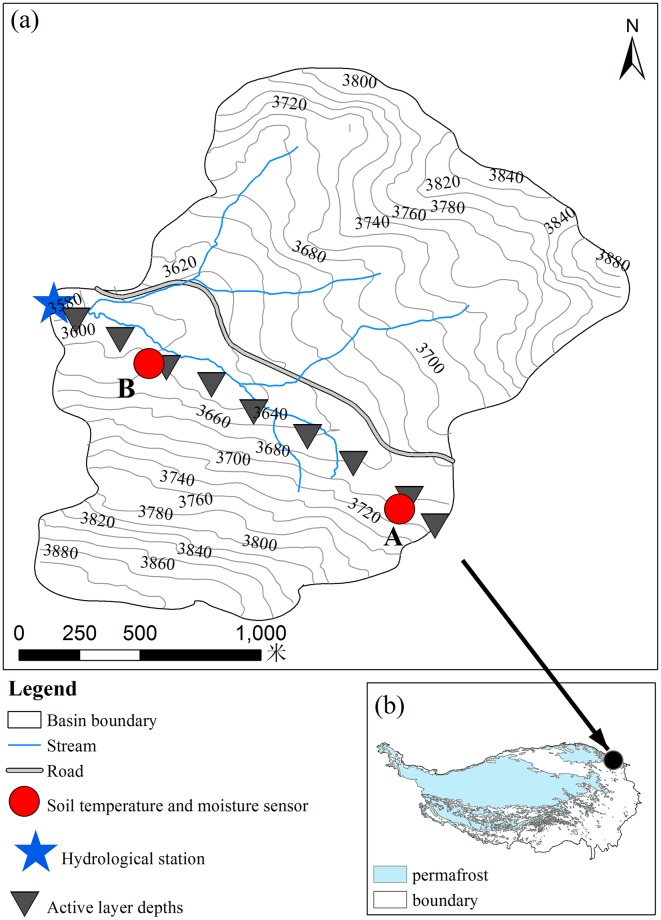
Permafrost hydrology observation network in the Eboling basin, at the headwaters of the Heihe River (a), and location on the Tibetan Plateau (b).

In this study, observations in the permafrost region of the Qilian-Altun Mountains in the northern Tibetan Plateau were conducted in 2014 and 2015 (Fig 1). This study describes: (1) characteristics of the active layer and (2) the runoff generation response to variations in thaw depth at the watershed scale in the northern Tibetan Plateau. Such local and regional studies are needed to increase understanding of the hydrological response to permafrost degradation in mid-latitude regions.

## Study description

### Study area

This study was carried out in the Qinghai-Tibet (Tibetan) Plateau with no specific permissions required for Chinese scientists. Field work procedure was in strict accordance with the environmental protection required by Lanzhou University. Field observation sites in our study did not involve endangered or protected species. The results will benefit local people and attract public attention to the cryospheric environmental conditions.

The study area is within the Qilian-Altun Permafrost Region of the Tibetan Plateau [20] an area of widespread mountain permafrost. The thickness of permafrost is ranging from 0–100 m. Permafrost temperature at depth of 19m was about -0.79°C as measured from a borehole drilled in 2011. Furthermore, ground ice and cryostructures were observed a few decimeters below the ground surface. This region is close to the Hexi Corridor, a string of oases along the northern edge of the Tibetan Plateau. The runoff from this permafrost region is used to support sustainable development of the environment, industry, and agriculture in arid and semiarid regions of Northwest China [1].

For this study, we investigated hydrological processes in the Eboling basin (lat. 37°59.5′–38°0.5′ N, lon. 100°54′–100°55′ E; alt. 3281–4263 m a.s.l.), a small alpine watershed in the Qilian Mountains with a drainage area of about 3 km^2^ (Fig 1). Wang et al. [21] and Mu et al. [22] previously investigated permafrost conditions and carbon properties in the Eboling basin. Soils in the Eboling basin are simple, consisting primarily of alpine cold desert soil, alpine steppe soil, and meadow soil. As a result of freezing and thawing, large areas of elevated mounds alternate with flatter and wetter depressions throughout the study area [23]. This land surface complicates the interface between the organic and mineral layers in the soil. The thickness of the peat layer varied between the two studied boreholes (Site A and B, Fig 1). Soil organic carbon (SOC) stocks in the active layer at Site A was 40.7 g m^–2^, and less than 47.8 g m^–2^ in the active layer at Site B. The depths of loam and sandy loam at Site A and Site B were approximately 30 cm and 40 cm, respectively. Based on the SOC content and texture in the soil profiles, the slope was covered unevenly by a continuous layer of organic soil of moderate thickness approximately 30–50 cm [22].

The vegetation cover was mainly alpine meadow and alpine steppe grasslands, dominated by sedges *Kobresia pygmaea*, *Kobresia tibetica*, and *Kobresia humilis*, and *Stipa purpurea* grass [24]. The region is characterized by an alpine semi-arid climate with an annual precipitation of approximately 430 mm, and an annual potential evaporation of about 1080 mm [25–26]. About 90% of precipitation falls as rain from May to September, with snowfall contributing less than 10% [25].

Mean annual air temperature ranges from –2.6 to –1.4°C [23]. Mean air temperature from May to September (summer) is 6.54 to 10.29°C, and –9.68 to –5.58°C in winter. Snow cover in this region has a negligible thermal effect on the permafrost because the snow cover is thin (<10 cm) [27].

### Field observation system

Table 1 summarized all data presented including details for equipment and measurement period. The hydrological station that monitored river water levels and flow was near the outlet of the Eboling basin, where two tributaries converge (Fig 1). On 15 May 2014, a Solinst automatic water level meter (Levelogger Model 3001 F2/M2, precision of ±0.1 cm) was installed in the still water well on the southeast side of the observation section in the hydrological station. The water level was recorded every 15 minutes and was calibrated using a Solinst pressure gauge (Barologger, precision of ±0.05 kPa). We calibrated the water level meter measurements by manually measuring water flow across the section using a propeller flow meter (LS1206B, Nanjing Automation Institute of Water Conservancy and Hydrology) every two weeks, and by calculating the streamflow using stage-discharge relations between the automatic water level record and the observed discharge. Precipitation was measured with a tipping bucket rainfall gauge (TE525MM, Campbell Scientific Inc.). The precipitation used the measured data rather than precipitation-correction data, due to the uncertainty factors of the precipitation correction [28–29]. Other meteorological parameters (temperature, humidity, and wind speed) were recorded every 30 minutes at a height of 2 m above the ground using an automatic weather station (CR1000, Campbell Scientific Inc.).

**Table 1:** List of equipment used, start of monitoring, time series and accuracy for each parameter in the Eboling basin, northern Tibetan Plateau.

Parameter	Equipment	Monitoring started	Time resolution	Accuracy
Water level	Solinst Levelogger Model 3001 Solinst pressure gauge	May 15, 2014	15 min	±0.1 cm ±0.05 kPa
Water flow	Nanjing Automation Institute of Water Conservancy and Hydrology, LS1206B	May, 2014	Two weeks 1 h (when hydrological events captured)	-
Precipitation	Campbell TE525MM	May, 2014	30 min	Up to 10 mm/hr ±1% 10 to 20 mm/hr 0~ –3% 20 to 30 mm/hr 0~ –5%
Soil temperature	Campbell 109 thermistor strings	May, 2014	30 min	±0.25°C
Soil moisture	Campbell CS616	May, 2014	30 min	±2.5%
Thaw depths	Mechanical probing	May, 2014	Two weeks	-

Soil temperature and moisture observations during from May 2014 to October 2015 were recorded every 30 minutes using an automatic data logger (CR1000, Campbell Scientific Inc.). Soil temperatures were measured by 109 thermistor strings (Campbell Scientific, Inc.) and soil volumetric (liquid) water contents were measured by CS616 time domain reflectometry (TDR) probes (Campbell Scientific, Inc.). The measurement accuracy of temperature and moisture sensor are ±0.25°C and ±2.5%, respectively. The temperature sensors and moisture sensors were inserted horizontally in the active layer and buried at depths of 5 cm, 10 cm, 20 cm, and 40 cm as a vertical array. The sensors were installed by digging a 0.8 m depth pit with a smooth, vertical wall to enable the horizontal insertion of the sensors into undisturbed soil. The pits were filled back with the removed soil in reverse order of removal, slightly compressed, and water saturated.

Thaw depths were measured at nine sites every two weeks by mechanical probing to the depth of resistance (Fig 1). The recorded depths represent the progression of the frost table from May 2014 to October 2015. This method has been used widely by the Circumpolar Active Layer Monitoring (CALM) program [30–31]. For each site, different surface conditions (e.g., depression, slope, and peat mound) were probed at least three times.

### Analytical procedure

A prolonged pause of the 0°C isotherm at a particular depth in freezing or thawing soils is known as the “zero curtain period” [32]. In ice-rich soils, much heat is consumed when converting water to ice and vice versa. This process gives rise to the zero-curtain effect and retards the rate of active layer freeze-thaw. In this study, we defined the duration of the zero curtain period using Quinton and Baltzer’s [7] method, i.e., the number of continuous cumulative days when daily temperature change at the depth of interest is <0.1°C. The soil temperature, soil moisture, thawing depth, and zero curtain period were used to analyze the characteristic of active layer in thawing period.

In the hydrological data, a runoff event was defined as a period of more than 2 h of discharge followed by at least 6 hours with no discharge. From 1 May to 31 October 2014, 31 runoff events were identified for further analysis for the hydrological properties:

1. Runoff trend, quantified using stream discharge and precipitation data.
2. Runoff lag, defined as the time difference between maximum precipitation rate and peak discharge at the hydrological gauge.
3. Runoff coefficients, defined for each runoff event by Eq (1):
  $$R_{c}=\frac{\int Qdt}{A\times P}$$ (1)
  where *R*_*c*_ is the runoff coefficient for each runoff event, *∫Qdt* is the total discharge that entered the stream on each runoff event (m^3^), *A* is the drainage area (m^2^), and *P* is the total precipitation before and during each runoff event (m).

In order to reflect the response of runoff to active layer characteristics, we analyzed the impact of variation of active layer characteristics on the runoff generation processes and the hydrological properties (i.e., runoff lag, runoff coefficient).

## Results

### Characteristics of active layer

**Fig 2:**
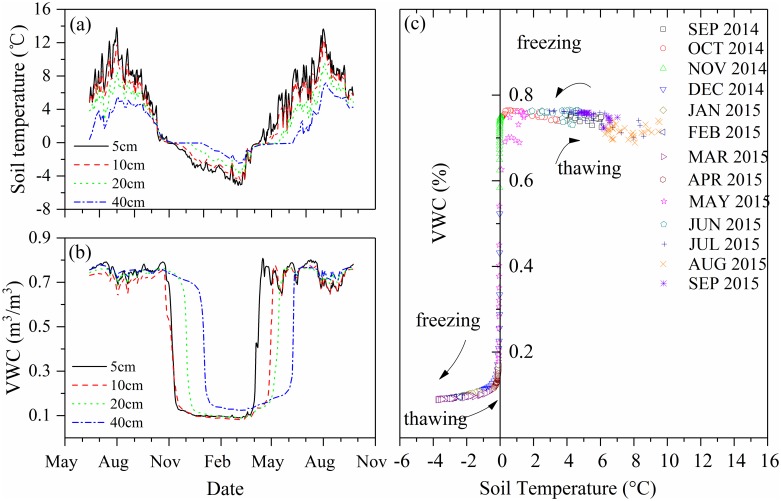
(a) Soil temperature and (b) unfrozen volumetric water content (VWC) at four selected depths (5, 10, 20, and 40 cm), and (c) VWC and soil temperature at 20 cm depth in 2014–2015.

Soil temperatures in 2014 and 2015 are shown in Fig 2a. The maximum and minimum daily soil temperatures occurred in August (13.82°C at 5 cm) and March (–5.10°C at 5 cm), respectively. There were five months (November to March) when the daily soil temperatures at all measured depths were below 0°C (Fig 2a). At the beginning of the zero curtain period during soil thawing, soil volumetric water content (VWC, m^3^/m^3^) reached and remained near saturation (0.76–0.77 m^3^/m^3^). In July and August, the unfrozen VWC fluctuated significantly below saturation. In early November, soil moisture rapidly decreased and the unfrozen VWC was only about 0.1 (Fig 2b).

Fig 2c shows the annual thawing and freezing cycle at 20 cm depth for 2014 and 2015. The duration of the zero-curtain at a depth of 20 cm was 54 days (24 October to 16 December 2014), during which time the VWC rapidly decreased from 0.76 to 0.12 m^3^/m^3^, and the average daily temperature decreased from 0.32 to –0.54°C. Between the minimum average daily temperature during the freezing period (–3.77°C) and the minimum temperature during the zero-curtain period (–0.54°C), the unfrozen VWC remained about 0.1. Once the temperature increased above –0.54°C, the ice in the soil began to melt and the active layer began thawing, returning the soil to a saturated state. However, when the soil started thawing following the zero-curtain period from 31 March to 15 May 2015, the VWC didn’t return to saturation immediately, but remained around 0.70 m^3^/m^3^ for a short period. The freezing process followed a similar pattern, whereby moisture remained in the thawed state until the minimum temperature during the zero-curtain period was reached. The largest change in VWC occurred in the narrow zero-curtain temperature range, 0.32 to –0.54°C. Further cooling after the zero-curtain period had a negligible impact on the VWC.

The duration of the freezing zero-curtain period increased with depth (Fig 2a and 2b). For the four depths between 0.05 m and 0.5 m, the duration increased from 16 to 113 days. During the soil thawing period, the duration of the zero-curtain also increased with depth. The duration of the zero-curtain was 11 days (30 March–10 April) at 5 cm depth, 27 days (31 March–27 April) at 10 cm depth, 44 days (31 March–14 May) at 20 cm depth, and 70 days (30 March–8 June) at 40 cm depth.

**Fig 3:**
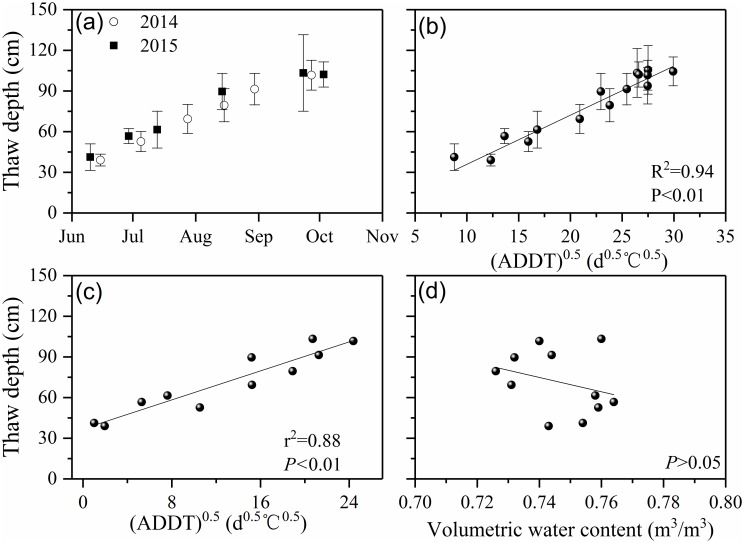
(a) Variability of thaw depths in summer, (b) thaw depth versus the square root of accumulated degree days of thaw (ADDT^0.5^) predicted by Stefan equation in summer 2014 and 2015 (bars indicate one standard deviation), thaw depth versus (c) ADDT^0.5^ of soil temperature and (d) unfrozen soil moisture at a depth of 20 cm.

Fig 3a shows temporal variations in average thaw depths during summer 2014 and 2015. At the end of the thawing season (early October), maximum thaw depth was up to 102±10 cm in the study area. There was a strong relation between thaw depth and accumulated degree-days of thaw (ADDT^0.5^) (r^2^ = 0.94, P<0.01, N = 15; Fig 3b), similar to that found in Arctic regions [33–35]. The relation between average thaw depth and the square root of ADDT (cumulative positive temperature) and soil moisture at 20 cm indicates significant correlation between thaw depth and ADDT^0.5^ (r^2^ = 0.88, p<0.01, Fig 3c), but no significant relation was found between thaw depth and moisture (Fig 3d).

Thaw depth is affected by a number of site factors including soil moisture, organic cover thickness, slope inclination, etc. [23, 36]. Observed average depth was 113±12 cm and 110±8 cm at peat mounds, and 86±24 cm and 95±20 cm at depressions, for Site A and B respectively (Fig 1). Due to the high soil moisture in depressions and the associated lateral water flow [37], the thaw depths in depressions were comparable at sites A and B, and were less than the thaw depths in peat mounds.

### Precipitation and discharge

**Fig 4:**
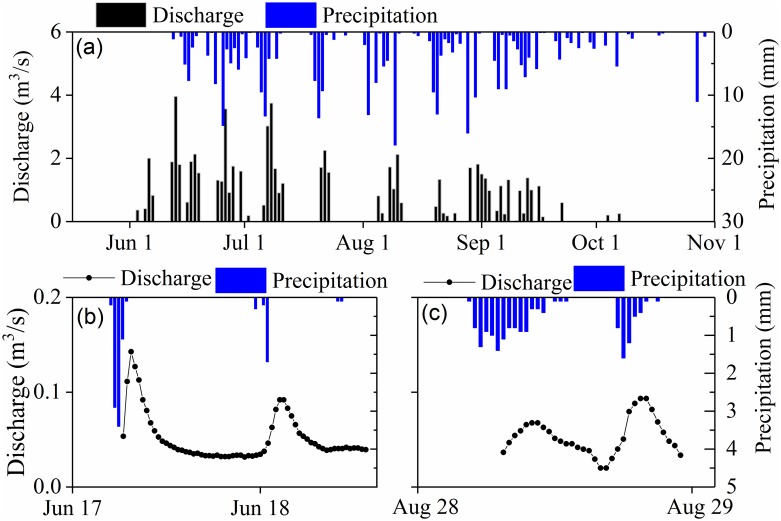
Time series of daily precipitation and streamflow (a), and precipitation and runoff events in 30 min intervals on (b) June 17 to 18 and (c) August 28.

Precipitation in the study area had a clear seasonal pattern, with wet periods in the summer, dry periods in winter, and little snowfall in spring and autumn (Fig 4a). The total measured precipitation at the Eboling basin in summer 2014 (13 June to 31 October) was 332 mm. Most precipitation occurred during several large monsoonal storms from late June to August, when the highest recorded precipitation rate was 17.9 mm/30 min. Fifty-eight percent of summer days had precipitation, but 70% of these days had less than 5 mm of rain, indicating that most precipitation events were small.

Runoff discharge did not persist throughout the summer. Intermittent flow was observed in the surface channel and along subsurface pathways only after some summer storms (Fig 4a). The response of runoff to precipitation was rapid; for example, the lag time between runoff and precipitation was only 1.5 h and 2 h on 17–18 June and 28 August, respectively (Fig 4b and 4c). There were 31 runoff generating events in the summer, with the longest persisting 96 h and the shortest lasting 2 h. Generally, these events were short, with 76% (23 events) lasting less than 24 h and 66% (16 events) lasting less than 12 h.

The thaw season in the Eboling basin lasted from early April to the end of October. However, the first runoff event did not occur until 3 June. This suggests that snow cover plays a negligible effect on runoff generation in the peat permafrost region. Even though there was some residual snow cover in early spring, most of this snow sublimated or melted and evaporated from the surface soil within a week [38](e.g., Bi et al., 2015). Due to limited snowmelt, the initiation of surface runoff was delayed in the river channel of peat permafrost in our study area.

**Fig 5:**
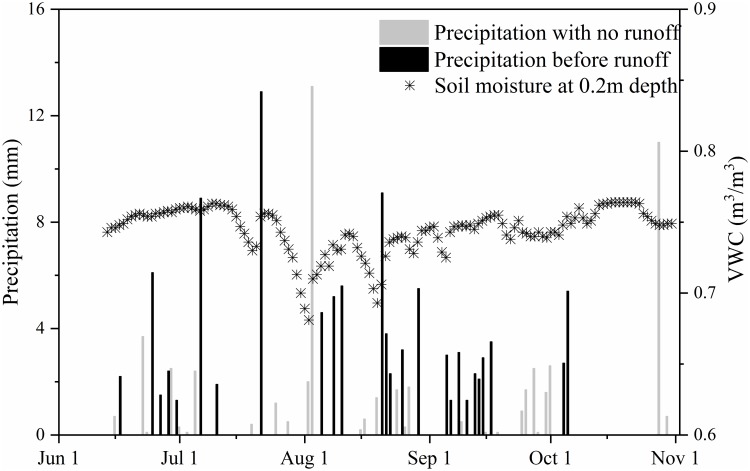
Precipitation events with and without runoff, and associated soil moisture content at 20 cm depth.

Runoff events measured at the Eboling gauge varied substantially over the summer and typically responded rapidly to precipitation. However, not all precipitation resulted in runoff. Fig 5 shows the relationship between precipitation with and without runoff yield and associated soil moisture at 20 cm depth. In 2014, 29 precipitation events did not yield runoff between 13 June to 31 October. Precipitation amounts for these events ranged from 0.1–13.1 mm, with 15 events having <1 mm and two having >10 mm. Precipitation before the events yielding runoff ranged from 1.3–12.9 mm, with 70% of events having 1–5 mm precipitation. Heavy precipitation events that did not yield runoff typically occurred when soil moisture was low. For instance, on 3 August, soil moisture at 20 cm depth was at a minimum value of 0.68. Precipitation on 19 August did not cause runoff because the thawed active layer could absorb up to 12.9 mm of precipitation. This indicates that snowmelt does not play a huge role, which would contrast it with sites in the Arctic and sub-Arctic for the most part.

## Discussion

### The active layer’s relationship with hydrothermal dynamics

The active layer dynamics in this study area are different from those in Arctic permafrost regions. In the northern Tibetan Plateau, the duration of soil thawing is longer than in Arctic areas such as Northern Canada [7], because the thin snow cover allows for earlier warming and thaw of the active layer [39]. Most parts of the Tibetan Plateau are influenced by a monsoon climate consistent with the soil thawing period in the Eboling basin. Frequent, small precipitation events accounting for most of the year’s rainfall reduce the surface radiation and ground surface temperature, and increase the latent heat flux [40]. The longer thawing period and deeper active layer in the northern Tibetan Plateau compared to the Arctic also results from higher air temperatures associated with the summer monsoonal climate at low latitudes [23, 36].

The surface peat layer is highly effective in reducing heat transfer to the soil, moderating ground warming in summer, and insulating the ground in winter. For example, the average daily soil temperature difference between 5 cm depth and 40 cm depth was 6.43°C in summer and 2.56°C in winter, respectively. The increasing duration of the zero-curtain at depth reflects the increasing moisture content with depth, where more latent energy is released to complete the freezing processes. This could explain why the average thaw depth (102±10 cm) in the Eboling basin was shallower than the active layer thickness along the Qinghai-Tibet Railway (218 cm) [41]. Soil along the railway is drier because water is drained by man-made structures. Furthermore, permafrost areas with ice-rich peat such as the Eboling basin have a longer zero-curtain period and require more heat to convert ice to water during soil thawing.

Soil moisture in the peat fluctuated significantly in the thawing period due to precipitation associated with monsoons. The saturated zone is bounded by the water table above, and the frost table below, and descends through the peat profiles as the ground thaws [7]. The frequent precipitation events in summer keep the soil saturated most of the time, however, the subsurface runoff and evaporation processes still cause soil moisture to decline significantly during long periods without rain. The distribution and duration of water pooled in depressions, which are also controlled by monsoon precipitation, significantly impact thaw depth. Thus, the monsoonal climate is the important factor controlling soil hydrothermal dynamics in the Tibetan Plateau.

### Runoff response to active layer development

**Fig 6:**
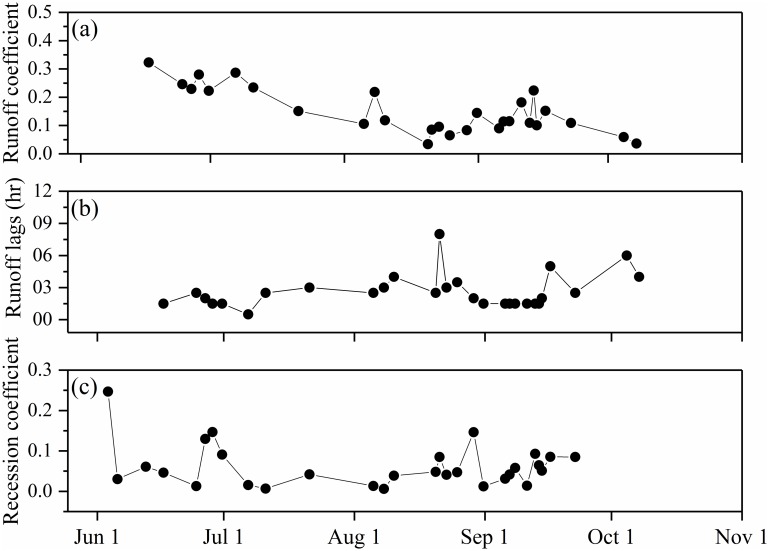
Hydrological properties during the thawing season: (a) runoff coefficient and (b) runoff lag coefficient.

With the active layer thawing upon catchment drainage, rapid runoff can be maintained by flow through shallow soil during early summer, and through the deeper preferential flow layer in the late summer [19, 42]. Although no direct measurements of preferential flow during the summer were made, the runoff lags and runoff coefficient may provide evidence of the development of soil pipes, a type of preferential flow mechanism in the catchment drainage [43–44]. The runoff coefficient indicated two distinct patterns: one before and one after mid-August (Fig 6a). The early stage showed that the partly-thawed active layer significantly retarded water storage, resulting in a high runoff coefficient. The flow was dominated by rapid runoff, which responded quickly to storms. As the active layer thawed, runoff generation decreased and the soil storage potential increased. The later stage revealed that active layer thaw had little influence on runoff generation, as runoff was dominated by the preferential flow in the deeper layer. Because of the frequent monsoon rainfall in July and August, active layer thaw continues until the plateau is transformed into a wetland (middle August in 2014), when the water table remains at or near the ground surface. The frequency of summer precipitation affects the saturated zone by changing the level of the water table.

Runoff lag indicated the delay between maximum precipitation rate and when surface runoff released to the outlet river. In this study, runoff lag varied little, except at the end of the thawing season, when it increased during several events (Fig 6b). Most events were characterized by maximum runoff that quickly decreased and then maintained a low level (about 0.03–5 cm^3^/s) until flow dissipated.

In this study area, runoff generation was most affected by active layer thawing, precipitation volumes, and the antecedent soil moisture conditions. Since permafrost is impervious and behaves as an aquiclude, transient saturation only occurred in the upper peat layer. Although soil moisture was high in peat, it varied greatly during the thawing season and was affected by precipitation frequency and volume. When the water content reached saturation, the elevation gradient exceeded the capillary potential and water “spilled” downslope over the saturated zone.

The peat runoff generation observed was similar to the fill and spill mechanism described by Tromp-van Meerveld and McDonnell for a hillslope [36, 45]. As the soil thaws and the depressions deepen, a larger amount of water is needed to fill them before the water stored upslope can connect to flow downslope [36]. Areas with limited thaw and the summer wetlands have a limited capacity to store water, so significant summer rainfall and a large hydraulic gradient cause excess water to flow into adjacent permafrost-free wetlands. Thus, in shallow soil, where thaw is limited and precipitation amounts are low, the duration of streamflow is transient.

## Conclusions

This study investigated the relation between active layer development and runoff processes in a mountainous permafrost region in the northern Tibetan Plateau by analyzing thaw depths, soil temperature, soil moisture, precipitation, and runoff.

The results indicate that the ice in the active layer in the northern Tibetan Plateau started to thaw when the soil temperature was greater than –0.54°C. In this permafrost region covered by peat, the active layer was shallower and the duration of the zero-curtain was longer than in other areas on the Tibetan Plateau, because more heat is needed to melt the large volume of ice in the porous surface organic material.

Runoff was generated intermittently, with the duration of most runoff events lasting less than 24 h. Because of the lack of snowmelt, the initiation of surface runoff was delayed in the peat material. Precipitation without runoff generation was generally correlated with lower soil moisture conditions. Earlier active layer thaw affected runoff generation capacity by decreasing the runoff coefficient. Active layer thaw continued until the plateau was transformed into a wetland, when the water table remained at or near the ground surface. The frequency of summer precipitation affected the saturation zone by changing the level of the water table. The active layer had to be saturated before runoff was generated. Thus, surface runoff was controlled by thaw depth, precipitation frequency and magnitude, and the antecedent soil hydrothermal properties.

## Supporting information

S1 Table List of equipment used, start of monitoring, time series and accuracy for each parameter. (DOCX)
