# Supplementary material for: Impacts of the active layer on runoff in an upland permafrost basin, northern Tibetan Plateau
Source: PLoS One. 2018 Feb 22;13(2):e0192591. doi: 10.1371/journal.pone.0192591 (PMC5823382; doi:10.1371/journal.pone.0192591)
Supplement: S1 Table — (DOCX) [file pone.0192591.s001.docx]

Supporting information

Impacts of the active layer on runoff in an upland permafrost basin, northern Tibetan Plateau

Tanguang Gao^1^, Tingjun Zhang^1^*, Hong Guo^2^, Yuantao Hu^1^, Jianguo Shang^1^, Yulan Zhang^3^*

^1^ Key Laboratory of Western China's Environmental Systems (Ministry of Education), College of Earth and Environmental Sciences, Lanzhou University, Lanzhou, 730000, China

^2^ University of Arkansas, Fayetteville, 72703, USA

^3^ State Key Laboratory of Cryospheric Science, Northwest Institute of Eco-Environment and Resources, Chinese Academy of Science, Lanzhou 730000, China

*Correspondence author

E-mail: Prof. Tingjun Zhang ([tjzhang@lzu.edu.cn](mailto:tjzhang@lzu.edu.cn)), and Dr. Yulan Zhang ([yulan.zhang@lzb.ac.cn](mailto:yulan.zhang@lzb.ac.cn))

**S1 Table.** Observation of soil temperature (°C) and soil moisture (m^3^/m^3^) in this study.

| Date | Soil Temperature | VWC |
| --- | --- | --- |
|  | °C | m^3^/m^3^ |
| 2014-6-14 | 0.743 | 3.824 |
| 2014-6-15 | 0.746 | 3.856 |
| 2014-6-16 | 0.746 | 3.958 |
| 2014-6-17 | 0.748 | 4.04 |
| 2014-6-18 | 0.749 | 4.249 |
| 2014-6-19 | 0.752 | 4.733 |
| 2014-6-20 | 0.754 | 4.225 |
| 2014-6-21 | 0.755 | 3.954 |
| 2014-6-22 | 0.756 | 3.837 |
| 2014-6-23 | 0.755 | 4.375 |
| 2014-6-24 | 0.754 | 4.818 |
| 2014-6-25 | 0.754 | 5.4 |
| 2014-6-26 | 0.756 | 4.827 |
| 2014-6-27 | 0.756 | 4.765 |
| 2014-6-28 | 0.757 | 5.708 |
| 2014-6-29 | 0.758 | 6.626 |
| 2014-6-30 | 0.757 | 6.74 |
| 2014-7-1 | 0.759 | 5.683 |
| 2014-7-2 | 0.76 | 5.186 |
| 2014-7-3 | 0.76 | 5.076 |
| 2014-7-4 | 0.761 | 4.74 |
| 2014-7-5 | 0.76 | 5.04 |
| 2014-7-6 | 0.759 | 5.305 |
| 2014-7-7 | 0.758 | 6.135 |
| 2014-7-8 | 0.759 | 5.91 |
| 2014-7-9 | 0.761 | 4.306 |
| 2014-7-10 | 0.763 | 2.74 |
| 2014-7-11 | 0.763 | 3.04 |
| 2014-7-12 | 0.762 | 3.308 |
| 2014-7-13 | 0.762 | 4.04 |
| 2014-7-14 | 0.761 | 4.185 |
| 2014-7-15 | 0.759 | 4.027 |
| 2014-7-16 | 0.754 | 4.083 |
| 2014-7-17 | 0.747 | 4.603 |
| 2014-7-18 | 0.742 | 5.497 |
| 2014-7-19 | 0.736 | 6.341 |
| 2014-7-20 | 0.73 | 6.908 |
| 2014-7-21 | 0.733 | 6.479 |
| 2014-7-22 | 0.754 | 5.801 |
| 2014-7-23 | 0.756 | 5.827 |
| 2014-7-24 | 0.756 | 5.695 |
| 2014-7-25 | 0.755 | 5.877 |
| 2014-7-26 | 0.751 | 6.023 |
| 2014-7-27 | 0.743 | 6.025 |
| 2014-7-28 | 0.737 | 6.726 |
| 2014-7-29 | 0.731 | 7.391 |
| 2014-7-30 | 0.725 | 7.45 |
| 2014-7-31 | 0.713 | 7.822 |
| 2014-8-1 | 0.7 | 8.37 |
| 2014-8-2 | 0.689 | 8.53 |
| 2014-8-3 | 0.681 | 8.02 |
| 2014-8-4 | 0.71 | 7.477 |
| 2014-8-5 | 0.713 | 6.768 |
| 2014-8-6 | 0.719 | 7.027 |
| 2014-8-7 | 0.727 | 6.698 |
| 2014-8-8 | 0.719 | 6.772 |
| 2014-8-9 | 0.734 | 6.936 |
| 2014-8-10 | 0.73 | 6.874 |
| 2014-8-11 | 0.731 | 7.292 |
| 2014-8-12 | 0.741 | 6.896 |
| 2014-8-13 | 0.742 | 6.274 |
| 2014-8-14 | 0.74 | 5.603 |
| 2014-8-15 | 0.732 | 5.266 |
| 2014-8-16 | 0.726 | 5.331 |
| 2014-8-17 | 0.721 | 5.81 |
| 2014-8-18 | 0.714 | 6.187 |
| 2014-8-19 | 0.703 | 6.584 |
| 2014-8-20 | 0.693 | 7.199 |
| 2014-8-21 | 0.706 | 7.398 |
| 2014-8-22 | 0.726 | 6.914 |
| 2014-8-23 | 0.736 | 6.565 |
| 2014-8-24 | 0.738 | 6.387 |
| 2014-8-25 | 0.739 | 6.118 |
| 2014-8-26 | 0.74 | 6.019 |
| 2014-8-27 | 0.739 | 5.927 |
| 2014-8-28 | 0.731 | 5.708 |
| 2014-8-29 | 0.728 | 5.719 |
| 2014-8-30 | 0.736 | 6.199 |
| 2014-8-31 | 0.744 | 5.724 |
| 2014-9-1 | 0.744 | 5.985 |
| 2014-9-2 | 0.746 | 5.797 |
| 2014-9-3 | 0.747 | 5.23 |
| 2014-9-4 | 0.739 | 5.233 |
| 2014-9-5 | 0.729 | 5.5 |
| 2014-9-6 | 0.725 | 6.058 |
| 2014-9-7 | 0.743 | 5.93 |
| 2014-9-8 | 0.747 | 5.453 |
| 2014-9-9 | 0.747 | 5.955 |
| 2014-9-10 | 0.748 | 5.862 |
| 2014-9-11 | 0.747 | 5.966 |
| 2014-9-12 | 0.748 | 5.701 |
| 2014-9-13 | 0.745 | 5.76 |
| 2014-9-14 | 0.749 | 6.036 |
| 2014-9-15 | 0.751 | 5.599 |
| 2014-9-16 | 0.753 | 5.013 |
| 2014-9-17 | 0.754 | 4.713 |
| 2014-9-18 | 0.755 | 4.176 |
| 2014-9-19 | 0.755 | 4.247 |
| 2014-9-20 | 0.749 | 4.26 |
| 2014-9-21 | 0.741 | 4.414 |
| 2014-9-22 | 0.738 | 4.768 |
| 2014-9-23 | 0.746 | 4.489 |
| 2014-9-24 | 0.751 | 3.945 |
| 2014-9-25 | 0.743 | 3.976 |
| 2014-9-26 | 0.742 | 4.099 |
| 2014-9-27 | 0.74 | 4 |
| 2014-9-28 | 0.74 | 3.875 |
| 2014-9-29 | 0.743 | 3.401 |
| 2014-9-30 | 0.74 | 3.412 |
| 2014-10-1 | 0.739 | 3.533 |
| 2014-10-2 | 0.743 | 3.39 |
| 2014-10-3 | 0.743 | 3.299 |
| 2014-10-4 | 0.741 | 3.05 |
| 2014-10-5 | 0.748 | 2.67 |
| 2014-10-6 | 0.754 | 2.396 |
| 2014-10-7 | 0.749 | 2.284 |
| 2014-10-8 | 0.753 | 2.279 |
| 2014-10-9 | 0.76 | 2.324 |
| 2014-10-10 | 0.753 | 2.498 |
| 2014-10-11 | 0.749 | 2.49 |
| 2014-10-12 | 0.751 | 1.918 |
| 2014-10-13 | 0.756 | 1.581 |
| 2014-10-14 | 0.762 | 1.294 |
| 2014-10-15 | 0.763 | 1.017 |
| 2014-10-16 | 0.763 | 0.799 |
| 2014-10-17 | 0.764 | 0.649 |
| 2014-10-18 | 0.764 | 0.597 |
| 2014-10-19 | 0.764 | 0.563 |
| 2014-10-20 | 0.764 | 0.654 |
| 2014-10-21 | 0.764 | 0.61 |
| 2014-10-22 | 0.764 | 0.474 |
| 2014-10-23 | 0.764 | 0.373 |
| 2014-10-24 | 0.763 | 0.318 |
| 2014-10-25 | 0.756 | 0.268 |
| 2014-10-26 | 0.754 | 0.216 |
| 2014-10-27 | 0.751 | 0.173 |
| 2014-10-28 | 0.749 | 0.14 |
| 2014-10-29 | 0.748 | 0.121 |
| 2014-10-30 | 0.748 | 0.105 |
| 2014-10-31 | 0.749 | 0.084 |
| 2014-11-1 | 0.749 | 0.067 |
| 2014-11-2 | 0.748 | 0.052 |
| 2014-11-3 | 0.748 | 0.041 |
| 2014-11-4 | 0.748 | 0.03 |
| 2014-11-5 | 0.748 | 0.023 |
| 2014-11-6 | 0.747 | 0.015 |
| 2014-11-7 | 0.746 | 0.008 |
| 2014-11-8 | 0.744 | 0.002 |
| 2014-11-9 | 0.743 | -0.002 |
| 2014-11-10 | 0.74 | -0.006 |
| 2014-11-11 | 0.738 | -0.008 |
| 2014-11-12 | 0.737 | -0.01 |
| 2014-11-13 | 0.734 | -0.013 |
| 2014-11-14 | 0.732 | -0.015 |
| 2014-11-15 | 0.729 | -0.016 |
| 2014-11-16 | 0.724 | -0.016 |
| 2014-11-17 | 0.721 | -0.019 |
| 2014-11-18 | 0.718 | -0.019 |
| 2014-11-19 | 0.714 | -0.021 |
| 2014-11-20 | 0.71 | -0.022 |
| 2014-11-21 | 0.705 | -0.023 |
| 2014-11-22 | 0.701 | -0.024 |
| 2014-11-23 | 0.694 | -0.022 |
| 2014-11-24 | 0.688 | -0.025 |
| 2014-11-25 | 0.681 | -0.024 |
| 2014-11-26 | 0.673 | -0.028 |
| 2014-11-27 | 0.663 | -0.026 |
| 2014-11-28 | 0.649 | -0.027 |
| 2014-11-29 | 0.625 | -0.025 |
| 2014-11-30 | 0.584 | -0.026 |
| 2014-12-1 | 0.523 | -0.026 |
| 2014-12-2 | 0.432 | -0.028 |
| 2014-12-3 | 0.334 | -0.032 |
| 2014-12-4 | 0.288 | -0.034 |
| 2014-12-5 | 0.256 | -0.042 |
| 2014-12-6 | 0.226 | -0.06 |
| 2014-12-7 | 0.209 | -0.083 |
| 2014-12-8 | 0.192 | -0.109 |
| 2014-12-9 | 0.171 | -0.144 |
| 2014-12-10 | 0.159 | -0.168 |
| 2014-12-11 | 0.149 | -0.211 |
| 2014-12-12 | 0.14 | -0.28 |
| 2014-12-13 | 0.133 | -0.361 |
| 2014-12-14 | 0.128 | -0.438 |
| 2014-12-15 | 0.126 | -0.47 |
| 2014-12-16 | 0.123 | -0.538 |
| 2014-12-17 | 0.12 | -0.661 |
| 2014-12-18 | 0.117 | -0.736 |
| 2014-12-19 | 0.117 | -0.763 |
| 2014-12-20 | 0.116 | -0.8 |
| 2014-12-21 | 0.115 | -0.827 |
| 2014-12-22 | 0.115 | -0.886 |
| 2014-12-23 | 0.114 | -0.937 |
| 2014-12-24 | 0.113 | -0.934 |
| 2014-12-25 | 0.113 | -0.963 |
| 2014-12-26 | 0.113 | -0.962 |
| 2014-12-27 | 0.113 | -0.966 |
| 2014-12-28 | 0.112 | -1.062 |
| 2014-12-29 | 0.11 | -1.183 |
| 2014-12-30 | 0.109 | -1.249 |
| 2014-12-31 | 0.108 | -1.308 |
| 2015-1-1 | 0.107 | -1.387 |
| 2015-1-2 | 0.107 | -1.404 |
| 2015-1-3 | 0.107 | -1.355 |
| 2015-1-4 | 0.107 | -1.351 |
| 2015-1-5 | 0.107 | -1.268 |
| 2015-1-6 | 0.107 | -1.205 |
| 2015-1-7 | 0.107 | -1.195 |
| 2015-1-8 | 0.107 | -1.272 |
| 2015-1-9 | 0.106 | -1.47 |
| 2015-1-10 | 0.104 | -1.641 |
| 2015-1-11 | 0.103 | -1.776 |
| 2015-1-12 | 0.102 | -1.848 |
| 2015-1-13 | 0.101 | -1.91 |
| 2015-1-14 | 0.101 | -1.938 |
| 2015-1-15 | 0.101 | -1.93 |
| 2015-1-16 | 0.1 | -1.989 |
| 2015-1-17 | 0.099 | -2.13 |
| 2015-1-18 | 0.099 | -2.129 |
| 2015-1-19 | 0.099 | -2.135 |
| 2015-1-20 | 0.098 | -2.181 |
| 2015-1-21 | 0.098 | -2.266 |
| 2015-1-22 | 0.097 | -2.335 |
| 2015-1-23 | 0.097 | -2.3 |
| 2015-1-24 | 0.097 | -2.184 |
| 2015-1-25 | 0.098 | -2.083 |
| 2015-1-26 | 0.098 | -2.063 |
| 2015-1-27 | 0.098 | -2.03 |
| 2015-1-28 | 0.098 | -1.955 |
| 2015-1-29 | 0.098 | -1.903 |
| 2015-1-30 | 0.098 | -2.001 |
| 2015-1-31 | 0.098 | -2.079 |
| 2015-2-1 | 0.098 | -2.096 |
| 2015-2-2 | 0.098 | -2.109 |
| 2015-2-3 | 0.097 | -2.178 |
| 2015-2-4 | 0.097 | -2.236 |
| 2015-2-5 | 0.097 | -2.289 |
| 2015-2-6 | 0.097 | -2.339 |
| 2015-2-7 | 0.097 | -2.377 |
| 2015-2-8 | 0.096 | -2.555 |
| 2015-2-9 | 0.095 | -2.723 |
| 2015-2-10 | 0.095 | -2.759 |
| 2015-2-11 | 0.095 | -2.745 |
| 2015-2-12 | 0.094 | -2.793 |
| 2015-2-13 | 0.094 | -2.839 |
| 2015-2-14 | 0.094 | -2.84 |
| 2015-2-15 | 0.094 | -2.79 |
| 2015-2-16 | 0.094 | -2.847 |
| 2015-2-17 | 0.093 | -2.974 |
| 2015-2-18 | 0.093 | -2.967 |
| 2015-2-19 | 0.094 | -2.743 |
| 2015-2-20 | 0.094 | -2.46 |
| 2015-2-21 | 0.096 | -2.09 |
| 2015-2-22 | 0.095 | -2.399 |
| 2015-2-23 | 0.094 | -2.85 |
| 2015-2-24 | 0.093 | -3.04 |
| 2015-2-25 | 0.093 | -3.055 |
| 2015-2-26 | 0.092 | -3.183 |
| 2015-2-27 | 0.092 | -3.304 |
| 2015-2-28 | 0.092 | -3.158 |
| 2015-3-1 | 0.091 | -3.316 |
| 2015-3-2 | 0.091 | -3.434 |
| 2015-3-3 | 0.09 | -3.532 |
| 2015-3-4 | 0.09 | -3.649 |
| 2015-3-5 | 0.091 | -3.208 |
| 2015-3-6 | 0.09 | -3.495 |
| 2015-3-7 | 0.09 | -3.556 |
| 2015-3-8 | 0.089 | -3.727 |
| 2015-3-9 | 0.089 | -3.684 |
| 2015-3-10 | 0.089 | -3.583 |
| 2015-3-11 | 0.091 | -3.02 |
| 2015-3-12 | 0.092 | -2.726 |
| 2015-3-13 | 0.092 | -2.743 |
| 2015-3-14 | 0.092 | -2.77 |
| 2015-3-15 | 0.09 | -3.442 |
| 2015-3-16 | 0.091 | -2.899 |
| 2015-3-17 | 0.093 | -2.13 |
| 2015-3-18 | 0.096 | -1.551 |
| 2015-3-19 | 0.098 | -1.221 |
| 2015-3-20 | 0.102 | -0.92 |
| 2015-3-21 | 0.1 | -1.206 |
| 2015-3-22 | 0.098 | -1.68 |
| 2015-3-23 | 0.097 | -1.878 |
| 2015-3-24 | 0.097 | -1.801 |
| 2015-3-25 | 0.096 | -1.996 |
| 2015-3-26 | 0.097 | -1.791 |
| 2015-3-27 | 0.101 | -1.155 |
| 2015-3-28 | 0.104 | -0.853 |
| 2015-3-29 | 0.107 | -0.669 |
| 2015-3-30 | 0.111 | -0.532 |
| 2015-3-31 | 0.114 | -0.441 |
| 2015-4-1 | 0.116 | -0.383 |
| 2015-4-2 | 0.119 | -0.342 |
| 2015-4-3 | 0.121 | -0.299 |
| 2015-4-4 | 0.123 | -0.269 |
| 2015-4-5 | 0.125 | -0.25 |
| 2015-4-6 | 0.127 | -0.234 |
| 2015-4-7 | 0.128 | -0.221 |
| 2015-4-8 | 0.129 | -0.214 |
| 2015-4-9 | 0.13 | -0.205 |
| 2015-4-10 | 0.131 | -0.197 |
| 2015-4-11 | 0.134 | -0.162 |
| 2015-4-12 | 0.135 | -0.149 |
| 2015-4-13 | 0.136 | -0.147 |
| 2015-4-14 | 0.136 | -0.148 |
| 2015-4-15 | 0.137 | -0.149 |
| 2015-4-16 | 0.138 | -0.149 |
| 2015-4-17 | 0.139 | -0.149 |
| 2015-4-18 | 0.14 | -0.147 |
| 2015-4-19 | 0.141 | -0.142 |
| 2015-4-20 | 0.143 | -0.141 |
| 2015-4-21 | 0.144 | -0.139 |
| 2015-4-22 | 0.145 | -0.136 |
| 2015-4-23 | 0.147 | -0.131 |
| 2015-4-24 | 0.149 | -0.121 |
| 2015-4-25 | 0.152 | -0.113 |
| 2015-4-26 | 0.155 | -0.107 |
| 2015-4-27 | 0.157 | -0.102 |
| 2015-4-28 | 0.16 | -0.097 |
| 2015-4-29 | 0.164 | -0.092 |
| 2015-4-30 | 0.17 | -0.089 |
| 2015-5-1 | 0.178 | -0.084 |
| 2015-5-2 | 0.185 | -0.082 |
| 2015-5-3 | 0.194 | -0.079 |
| 2015-5-4 | 0.205 | -0.074 |
| 2015-5-5 | 0.222 | -0.074 |
| 2015-5-6 | 0.255 | -0.07 |
| 2015-5-7 | 0.283 | -0.069 |
| 2015-5-8 | 0.306 | -0.067 |
| 2015-5-9 | 0.321 | -0.064 |
| 2015-5-10 | 0.35 | -0.062 |
| 2015-5-11 | 0.377 | -0.059 |
| 2015-5-12 | 0.402 | -0.056 |
| 2015-5-13 | 0.45 | -0.054 |
| 2015-5-14 | 0.541 | -0.044 |
| 2015-5-15 | 0.626 | 0.082 |
| 2015-5-16 | 0.691 | 0.298 |
| 2015-5-17 | 0.701 | 0.5 |
| 2015-5-18 | 0.701 | 0.68 |
| 2015-5-19 | 0.694 | 0.919 |
| 2015-5-20 | 0.689 | 1.164 |
| 2015-5-21 | 0.731 | 1.098 |
| 2015-5-22 | 0.748 | 0.575 |
| 2015-5-23 | 0.757 | 1.396 |
| 2015-5-24 | 0.758 | 1.567 |
| 2015-5-25 | 0.759 | 1.319 |
| 2015-5-26 | 0.755 | 1.337 |
| 2015-5-27 | 0.748 | 1.413 |
| 2015-5-28 | 0.752 | 1.438 |
| 2015-5-29 | 0.761 | 1.009 |
| 2015-5-30 | 0.762 | 1.239 |
| 2015-5-31 | 0.758 | 2.957 |
| 2015-6-1 | 0.758 | 2.386 |
| 2015-6-2 | 0.758 | 2.704 |
| 2015-6-3 | 0.758 | 2.803 |
| 2015-6-4 | 0.761 | 1.808 |
| 2015-6-5 | 0.763 | 1.919 |
| 2015-6-6 | 0.763 | 2.503 |
| 2015-6-7 | 0.762 | 2.531 |
| 2015-6-8 | 0.761 | 2.807 |
| 2015-6-9 | 0.757 | 3.154 |
| 2015-6-10 | 0.756 | 3.867 |
| 2015-6-11 | 0.754 | 3.892 |
| 2015-6-12 | 0.745 | 3.716 |
| 2015-6-13 | 0.736 | 4.248 |
| 2015-6-14 | 0.732 | 4.221 |
| 2015-6-15 | 0.737 | 3.769 |
| 2015-6-16 | 0.73 | 4.342 |
| 2015-6-17 | 0.751 | 4.773 |
| 2015-6-18 | 0.759 | 4.598 |
| 2015-6-19 | 0.761 | 4.556 |
| 2015-6-20 | 0.763 | 4.508 |
| 2015-6-21 | 0.765 | 4.146 |
| 2015-6-22 | 0.764 | 4.669 |
| 2015-6-23 | 0.764 | 4.261 |
| 2015-6-24 | 0.764 | 4.002 |
| 2015-6-25 | 0.763 | 4.495 |
| 2015-6-26 | 0.764 | 3.711 |
| 2015-6-27 | 0.763 | 3.854 |
| 2015-6-28 | 0.762 | 4.48 |
| 2015-6-29 | 0.763 | 4.685 |
| 2015-6-30 | 0.764 | 4.562 |
| 2015-7-1 | 0.764 | 4.528 |
| 2015-7-2 | 0.762 | 4.875 |
| 2015-7-3 | 0.761 | 4.901 |
| 2015-7-4 | 0.762 | 3.806 |
| 2015-7-5 | 0.762 | 3.203 |
| 2015-7-6 | 0.763 | 2.941 |
| 2015-7-7 | 0.761 | 3.841 |
| 2015-7-8 | 0.76 | 4.223 |
| 2015-7-9 | 0.761 | 4.163 |
| 2015-7-10 | 0.764 | 3.262 |
| 2015-7-11 | 0.761 | 4.263 |
| 2015-7-12 | 0.757 | 4.615 |
| 2015-7-13 | 0.759 | 5.971 |
| 2015-7-14 | 0.758 | 6.265 |
| 2015-7-15 | 0.756 | 5.608 |
| 2015-7-16 | 0.754 | 5.152 |
| 2015-7-17 | 0.759 | 4.883 |
| 2015-7-18 | 0.761 | 5.068 |
| 2015-7-19 | 0.759 | 4.691 |
| 2015-7-20 | 0.756 | 4.405 |
| 2015-7-21 | 0.748 | 4.671 |
| 2015-7-22 | 0.741 | 5.08 |
| 2015-7-23 | 0.74 | 5.585 |
| 2015-7-24 | 0.749 | 5.461 |
| 2015-7-25 | 0.734 | 6.085 |
| 2015-7-26 | 0.724 | 6.735 |
| 2015-7-27 | 0.712 | 7.239 |
| 2015-7-28 | 0.7 | 7.994 |
| 2015-7-29 | 0.704 | 8.58 |
| 2015-7-30 | 0.741 | 8.29 |
| 2015-7-31 | 0.729 | 8.37 |
| 2015-8-1 | 0.713 | 9.07 |
| 2015-8-2 | 0.715 | 9.47 |
| 2015-8-3 | 0.737 | 9.54 |
| 2015-8-4 | 0.74 | 9.46 |
| 2015-8-5 | 0.731 | 8.7 |
| 2015-8-6 | 0.718 | 8.86 |
| 2015-8-7 | 0.713 | 8.62 |
| 2015-8-8 | 0.712 | 8.47 |
| 2015-8-9 | 0.712 | 7.946 |
| 2015-8-10 | 0.705 | 7.987 |
| 2015-8-11 | 0.699 | 7.983 |
| 2015-8-12 | 0.696 | 7.673 |
| 2015-8-13 | 0.689 | 8.06 |
| 2015-8-14 | 0.707 | 7.839 |
| 2015-8-15 | 0.732 | 6.935 |
| 2015-8-16 | 0.716 | 7.201 |
| 2015-8-17 | 0.723 | 7.455 |
| 2015-8-18 | 0.741 | 6.756 |
| 2015-8-19 | 0.729 | 6.768 |
| 2015-8-20 | 0.721 | 6.742 |
| 2015-8-21 | 0.72 | 6.516 |
| 2015-8-22 | 0.718 | 6.453 |
| 2015-8-23 | 0.713 | 6.474 |
| 2015-8-24 | 0.709 | 6.29 |
| 2015-8-25 | 0.7 | 6.65 |
| 2015-8-26 | 0.697 | 6.775 |
| 2015-8-27 | 0.696 | 6.709 |
| 2015-8-28 | 0.714 | 7.103 |
| 2015-8-29 | 0.736 | 6.457 |
| 2015-8-30 | 0.724 | 6.691 |
| 2015-8-31 | 0.721 | 6.43 |
| 2015-9-1 | 0.722 | 6.221 |
| 2015-9-2 | 0.725 | 6.164 |
| 2015-9-3 | 0.736 | 6.566 |
| 2015-9-4 | 0.746 | 6.551 |
| 2015-9-5 | 0.748 | 6.493 |
| 2015-9-6 | 0.746 | 6.486 |
| 2015-9-7 | 0.741 | 6.573 |
| 2015-9-8 | 0.748 | 7.026 |
| 2015-9-9 | 0.751 | 6.355 |
| 2015-9-10 | 0.754 | 5.569 |
| 2015-9-11 | 0.755 | 5.328 |
| 2015-9-12 | 0.757 | 5.048 |
| 2015-9-13 | 0.757 | 5.025 |
| 2015-9-14 | 0.758 | 4.722 |
| 2015-9-15 | 0.758 | 4.837 |
| 2015-9-16 | 0.758 | 4.966 |
| 2015-9-17 | 0.758 | 5.152 |
| 2015-9-18 | 0.758 | 5.225 |
| 2015-9-19 | 0.759 | 5.293 |
| 2015-9-20 | 0.759 | 5.18 |
| 2015-9-21 | 0.759 | 5.224 |
| 2015-9-22 | 0.759 | 5.22 |
| 2015-9-23 | 0.76 | 4.833 |
